# The efficacy and safety of anti-PD-1/PD-L1 antibodies for treatment of advanced or refractory cancers: a meta-analysis

**DOI:** 10.18632/oncotarget.12230

**Published:** 2016-09-24

**Authors:** Tengfei Zhang, Jing Xie, Seiji Arai, Liping Wang, Xuezhong Shi, Ni Shi, Fen Ma, Sen Chen, Lan Huang, Li Yang, Wang Ma, Bin Zhang, Weidong Han, Jianchuan Xia, Hu Chen, Yi Zhang

**Affiliations:** ^1^ Biotherapy Center, Cancer Center, The First Affiliated Hospital of Zhengzhou University, Zhengzhou, Henan, China; ^2^ Department of Hematology and Oncology, Beth Israel Deaconess Medical Center, Harvard Medical School, Boston, Massachusetts, United States; ^3^ School of Public Health, Xinxiang Medical University, Xinxiang, Henan, China; ^4^ Department of Urology, Gunma University Graduate School of Medicine, 3-39-22 Showa-machi, Maebashi, Japan; ^5^ Department of Oncology, the First Affiliated Hospital of Zhengzhou University, Zhengzhou, Henan, China; ^6^ Department of Epidemiology and Biostatistics, School of Public Health, Zhengzhou University, Zhengzhou, Henan, China; ^7^ Comprehensive Cancer Center, the Ohio State University, Columbus, Ohio, United States; ^8^ Department of Hematopoietic Stem Cell Transplantation, Affiliated Hospital to Academy of Military Medical Science (307 Hospital, PLA), Beijing, China; ^9^ Molecular and Immunological/Bio-therapeutic Department, Institute of Basic Medicine, Chinese PLA General Hospital, Beijing China; ^10^ Biotherapy Center, Cancer Center, Sun Yat-sen University, Guangzhou, Guangzhou, Guangdong, China; ^11^ Henan Key Laboratory for Tumor Immunology and Biotherapy, Henan, China; ^12^ School of Life Sciences, Zhengzhou University, Zhengzhou, Henan, China

**Keywords:** PD-1, PD-L1, immunotherapy, advanced or refractory cancer, meta-analysis

## Abstract

**Purpose:**

To systematically evaluate the overall efficacy and safety of current anti-PD-1/PD-L1 antibodies for treatment of patients with advanced or refractory cancer.

**Results:**

Fifty-one trials including 6,800 patients were included. The overall response rates for melanoma, non-small cell lung cancer (NSCLC), and renal cell carcinoma (RCC) were 29% (95% CI: 1.53−2.41), 21% (95% CI: 17%−25%) and 21% (95% CI: 16%−27%) respectively. While the overall adverse effects rate for melanoma, NSCLC, RCC were 16% (95% CI: 6%−28%), 11% (95% CI: 8%−14%) and 20% (95% CI: 11%−32%) respectively. Tumor PD-L1 expression and patient smoking status might serve as biomarkers to predict response of anti-PD-1/PD-L1 antibody treatment. Compared to tumors with negative PD-L1 expression, tumors with positive PD-L1 expression had a significantly higher clinical response rate (41.4% versus 26.5%) with RR = 1.92 (95% CI: 1.53−2.41, *P* < 0.001). Smoker patients also showed a significantly higher response rate (33.7%) than patients who never smoked (4.2%) with RR = 6.02 (95% CI: 1.22−29.75, *P* = 0.028). Nivolumab and Pembrolizumab were associated with significantly increased response rate (RR = 2.89, 95% CI: 2.46−3.40, *P* < 0.001), reduced death risk (HR= 0.53; 95% CI: 0.48−0.57; *P* < 0.001), and decreased adverse effect rate (RR = 0.49, 95% CI: 0.30−0.80, *P* = 0.004) compared with other therapies.

**Experimental Design:**

Clinical trials reporting response or safety of anti-PD-1/PD-L1 antibodies for advanced or refractory cancer patients published before January 31th 2016 were searched in PubMed and EMBASE database. Meta-analyses using random effects models were used to calculate the overall estimate.

**Conclusions:**

Anti-PD-1/PD-L1 antibodies have high response rates and low adverse effect rates for advanced or refractory cancers.

## INTRODUCTION

Cancer is still one of the most pressing health issues worldwide [1]. Although surgery, radiation and chemotherapy have significantly improved the clinical benefits for patients with localized cancer, therapies for advanced or refractory cancer patients still present a challenge [2]. Patients with advanced cancer usually miss the opportunity for surgery due to late diagnosis. Because of the diversities of genetic and epigenetic mutations harbored by cancer cells, a subpopulation of those patients rarely benefit from conventional systematic chemotherapy [2, 3]. In addition, immune evasion by cancer cells that spoil the initiation of effective antitumor response in cancer microenvironment inevitably presents problems for treatment of advanced and refractory cancer patients [3, 4]. However, endogenous immune response generated by “immune checkpoint” inhibitors was beneficial for cancer regression. The persistent immune response and effective immunologic memory might also have enabled the sustained control of tumor growth. Therefore immunotherapy, especially immunotherapy based on “immune checkpoint” inhibitors, showed broad advantages with durable clinical responses for advanced or refractory cancer patients [5].

Programmed death 1 (PD-1) is a highly expressed immune checkpoint receptor in immune lymphocytes. PD-1 is normally required for limiting autoimmunity and modulating the strength of T cell response in peripheral infected tissues in order to minimize damage to surrounding normal tissues [5, 6]. This inhibitory effect on immune activation is executed by the interaction of PD-1 with its ligand PD-L1. Importantly, PD-1 is highly expressed on tumor-infiltrating lymphocytes, whereas PD-L1 is upregulated in many human cancer cells so that cancers can escape from the immunologic surveillance by suppressing the immune function of T cells [7]. Therefore, antibodies against PD-1 and PD-L1 are promising therapies since they can reactivate the patient's own immune system, especially lymphocytes in tumor microenvironment, to fight against cancers by maintaining T cell activation.

Clinical trials have shown that anti-PD-1 antibodies Pembrolizumab and Nivolumab had promising overall response rate and prolonged survival for advanced or refractory melanoma patients [8, 9]. In one of the Pembrolizumab trial, the overall response rate was 26% and the estimated overall survival at 1 year was 63% in patients who received 10 mg/kg Pembrolizumab [8]. In another randomized open-label trial to compare Nivolumab with chemotherapy, the overall response rate was 31.7% in the Nivolumab group, compared to 10.6% in the chemotherapy group [9]. Therefore, the US Food and Drug Administration approved them for treatment of patients with unresectable or metastatic melanoma and disease progression following Ipilimumab (anti-CTL4 antibody), and for B-Raf proto-oncogene, serine/threonine kinase (BRAF) V600 mutation-positive patients with a BRAF inhibitor respectively. The improved response and prolonged survival of anti-PD-1/PD-L1 antibodies were also supported by other trials for patients with metastatic non-small cell lung cancer (NSCLC), hematological malignancies, renal cell cancer (RCC), bladder cancer, colon cancer and some other cancers [10–20]. However, systematic evaluation of the overall efficiency of anti-PD-1/PD-L1 antibodies for advanced or refractory cancer patients was limited. The published studies only summarized data for one or few types of cancers [21, 22]. Moreover, the key factors associated with better clinical responses still remain unclear. Thus, we will systematically evaluate the efficiency and safety of current clinical anti-PD-1/PD-L1 antibodies for advanced or refractory cancers and use this data to investigate potential factors associated with clinical responses. This study will present comprehensive data and evidence for future clinical application of anti-PD-1/PD-L1 antibodies based on current clinical trials.

## RESULTS

### Summary of included studies

**Figure 1:**
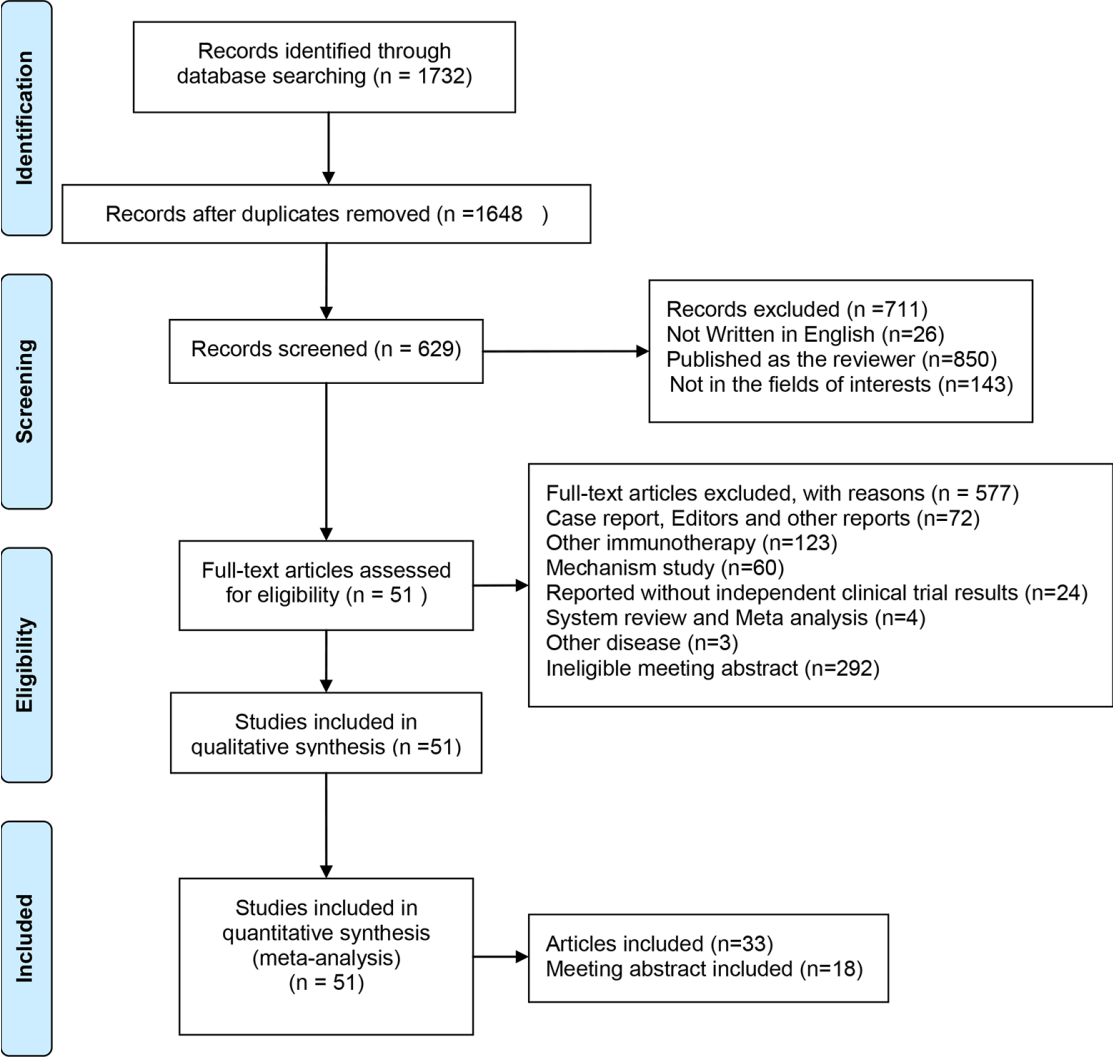
Flow diagram of study selection process

After removing duplicated literatures, unrelated literatures and some ineligible literatures, two investigators identified articles eligible for further review by screening titles and abstracts. We identified 33 literatures and 18 conference abstracts with a total of 6,800 patients involved (Figure 1). Both solid cancer patients and hematologic malignancy patients participated in these trials. Within these trials, data of 6,160 patients were used for efficiency analysis and and 6,273 patients for safety analysis. All the patients that received PD-1/PD-L1 antibodies were in advanced stages and most of the patients received previous treatment. The antibodies used in these trials included the anti-PD-1 antibodies Nivolumab, Pidilizumab and Pembrolizumab; and the anti-PD-L1 antibodies: BMS-936559, MPDL3280A and Avelumab. All the information of the clinical trials is listed in Supplementary Table S1. Literature quality assessment by MINORS showed that literatures recruited in this study were in good quality (Supplementary Table S2).

### Overall response rate

Meta-analysis showed that the overall pooled response rates of anti-PD-1/PD-L1 antibodies was 24% (95% CI: 21%–28%) in cancer patients with advanced stage, refractory or sensitive to previous treatment (Table 1 and Supplementary Figure S1A). Egger's regression asymmetry test (*P* = 0.105) showed no evidence of substantial publication bias and the funnel plot is listed in Supplementary Figure S2. Univariate meta-regression analysis showed that NSCLC, combination and antigen origin positively associated with anti-PD-1/PD-L1 antibody responses. Subgroup analyses also pooled the response rate for each drug and tumors (Table 1, Supplementary Figure S1B and S1C). The FDA approved anti-PD-1 antibodies, Nivolumab and Pembrolizumab showed promising response rates at 27% (95% CI: 21%–33%, Z = 14.61, *P* < 0.001) and 26% (95% CI: 21%–31%, Z = 15.64, *P* < 0.001) respectively. The pooled response rates for melanoma, NSCLC, RCC were 29% (95% CI: 23%–36%, Z = 14.70, *P* < 0.001), 21% (95% CI: 17%–25%, Z = 16.16, *P* < 0.001) and 21% (95% CI: 16%–27%, Z = 11.88, *P* < 0.001) respectively.

**Table 1:** Meta-regression analysis for response rates and adverse effect rates of anti-PD-1/PD-L1 antibodies in cancers

Factors at study level	Response rates						Adverse effects rates					
	No. of Data^a^	Pooled Response Rate (95% CI)	*I*^2^ (%)	*P* for *I*^2^	Meta−regression analysis		No. of Data *	Pooled Response Rate (95% CI)	*I*^2^ (%)	*P* for *I*^2^	Meta−regression analysis	
					Coefficients (95% CI)	*P* value					Coefficients (95% CI)	*P* value
Tumor type												
Melanoma	19	0.29 (0.23, 0.36)	91.22	< 0.001	0		13	0.16 (0.06, 0.28)	97.97	< 0.001	0	
NSCLC	14	0.21 (0.17, 0.25)	73.51	< 0.001	−0.12 (−0.20, −0.03)	0.008	8	0.11 (0.08, 0.14)	62.24	0.01	−0.07 (−0.21, 0.08)	0.363
RCC	9	0.21 (0.16, 0.27)	73.00	< 0.001	−0.10 (−0.21, −0.00)	0.046	7	0.20 (0.11, 0.32)	92.51	< 0.001	0.03 (−0.13, 0.18)	0.708
Hematologic malignancies	4	0.54 (0.20, 0.86)	94.96	< 0.001	0.11 (−0.07, 0.29)	0.230	4	0.30 (0.03, 0.69)	94.71	< 0.001	0.19 (−0.05, 0.43)	0.121
Ovarian cancer	3	0.14 (0.07, 0.24)	0.000	0.85	−0.18 (−0.44, 0.08)	0.163	2	0.16 (0.06, 0.29)	98.43	< 0.001	0.02 (−0.34, 0.38)	0.907
Bladder cancer	2	0.26 (0.17, 0.35)	0.000	0.57	−0.07 (−0.31, 0.17)	0.560	2	0.09 (0.04, 0.15)	98.75	< 0.001	−0.09 (−0.38, 0.20)	0.525
Esophageal cancer	1	0.22 (0.07, 0.44)	NA	NA	−0.11 (−0.55, 0.33)	0.619	1	0.26 (0.10, 0.48)	NA	NA	0.08 (−0.42, 0.58)	0.751
Mixed cancer^b^	6	0.09 (0.02, 0.19)	74.19	< 0.001	−0.22 (−0.39, −0.06)	0.009	6	0.14 (0.06, 0.24)	92.14	< 0.001	−0.03 (−0.20, 0.13)	0.702
Drug												
Nivolumab	28	0.27 (0.21, 0.33)	92.39	< 0.001	0		23	0.18 (0.11, 0.26)	96.98	< 0.001	0	
Pembrolizumab	14	0.26 (0.21, 0.31)	75.04	< 0.001	−0.02 (−0.11, 0.07)	0.954	11	0.09 (0.06, 0.14)	81.36	< 0.001	−0.08 (−0.21, 0.05)	0.204
MPDL3280A	8	0.18 (0.15, 0.22)	4.92	0.39	−0.10 (−0.22, 0.02)	0.087	4	0.13 (0.10, 0.16)	0.00	0.45	−0.06 (−0.23, 0.11)	0.494
BMS−936559	4	0.10 (0.03, 0.18)	51.17	0.10	−0.18 (−0.37, 0.01)	0.056	4	0.31 (0.03, 0.70)	94.61	< 0.001	0.19 (−0.04, 0.42)	0.110
Pidilizumab	3	0.37 (0.03, 0.82)	96.70	< 0.001	0.01 (−0.18, 0.21)	0.882	1	0.09 (0.06, 0.14)	NA	NA	−0.10 (−0.40, 0.19)	0.490
Avelumab	1	0.17 (0.05, 0.39)	NA	NA	NA	NA	1	NA	NA	NA	NA	NA
Combination												
No Combination	49	0.21 (0.18, 0.24)	82.43	< 0.001	0	< 0.001	35	0.13 (0.10, 0.16)	86.94	< 0.001	0	< 0.001
Combination	9	0.41 (0.32, 0.51)	85.66	< 0.001	−0.22 (−0.30, −0.15)		8	0.29 (0.14, 0.46)	95.30	< 0.001	0.29 (0.18, 0.40)	
Antigen Origin												
Anti−PD−L1	13	0.16 (0.12, 0.20)	39.57	0.07	0	0.018	38	0.17 (0.11, 0.22)	96.10	< 0.001	0	0.439
Anti−PD−1	45	0.27 (0.23, 0.31)	90.83	< 0.001	−0.12 (−0.22, −0.02)		5	0.12 (0.09, 0.15)	24.51	0.26	−0.06 (−0.21, 0.09)	

a the number of data set extracted from studies

b Mix cancer: multiple tumors included in these four studies but can't be separately totally by tumor types.

### Anti-PD-1/PD-L1 antibodies have higher clinical response rates than regular chemotherapy and Ipilimumab in melanoma patients

**Figure 2:**
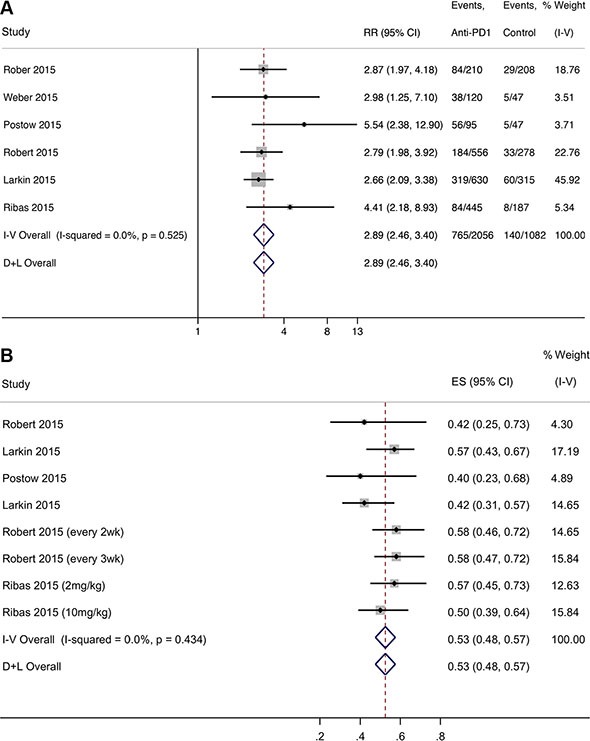
Forest plot for ratio risk and confidence intervals of response rate (A) and PFS survival (B) of anti-PD-1 antibody compared with other therapies for melanoma

We compared the response rates of anti/PD-1/PD-L1 antibodies (Nivolumab and Pembrolizumab) with other regular chemotherapy and Ipilimumab in melanoma patients (6 studies, details see Supplementary Table S1). We found that anti-PD-1/PD-L1 antibodies were associated with a significant increase in the response rates compared with other therapies (RR = 2.89, 95% CI: 2.46–3.40, *P* < 0.001) with no evidence of heterogeneity (*I*^2^ = 0.00, χ^2^ = 4.17, *P* = 0.525) (Figure 2A). Begg's regression asymmetry test (*P* = 0.06) showed no evidence of substantial publication bias. Compared to the control group, where 129 people out of 1000 had response events, 372 out of 1000 treated with the anti-PD-1/PD-L1 antibodies had response cases. Based on a rate of 12.9%, the NNTB would be 4. Compared to other therapies, the number of response cases added per 1000 individuals by anti-PD-1/PD-L1 drugs was 243. Nivolumab alone was associated with a significant increase in the response rate compared to other therapies (4 studies, RR = 2.83, 95% CI: 2.34–3.43, *P* < 0.001), with no evidence of heterogeneity (*I*^2^ = 0.00, χ ^2^= 2.70, *P* = 0.439). Pembrolizumab was also associated with a significant increase in the response rate compared to other therapies (2 studies, RR = 3.04, 95% CI: 2.24–4.13, *P* < 0.001), with slight heterogeneity (*I*^2^ = 24.3, χ^2^ = 1.32, *P* = 0.251, Supplementary Figure S1D). Moreover, these two anti-PD-1 antibodies (Nivolumab and Pembrolizumab) substantially reduced the risk of death compared with other therapies (8 studies, HR = 0.53; 95% CI: 0.48–0.57; *P* < 0.001), with no evidence of heterogeneity (*I*^2^ = 0.00, χ^2^ = 6.95, *P*=4.34, Figure 2B).

### Potential biomarkers to predict clinical outcomes

We evaluated the current reported factors that might potentially affect the clinical outcomes in different cancers. These potential factors include PD-L1 expression, tumor-infiltrating lymphocytes, gene mutation (BRAF, EGFR, KRAS and DNA mismatch repair related genes), blood immune biomarkers (such as IL-18, ITAC, IFN-γ, IL-6) and patients' life style such as smoking history, etc. The details of these factors are listed in Table 2.

**Table 2:** Potential biomarkers for predicting clinical outcomes of PD-1/PD-L1 antibody application

	Melanoma	NSCLC	RCC	Hematologic malignancies	Others solid tumor (Colon, bladder, etc)
1. PD1/PD-L1 signaling pathway	PD-L1 expression on tumor cells (9)	PD-L1 expression on tumor cells (6) and TIL(1)	PD-L1 expression on tumor cells (2)	PD-L1, PD-L2 copy number (1) and PD-L1 expression(1)	PD-L1 expression on tumor cells (2) and TIL (1)
2. Genes mutation	BRAF (3)	EGFR(2), KRAS(2)	NA	NA	Mismatch repair status (1)
3. Lymphocytes subgroup	CD25^+^Treg/CD4^+^T-cell ratio(1), MDSC (1), CD8 T cell (1), T_regs_(CD4^+^ CD25^+^ CD127^low^FoxP3^+^) (1), Absolute lymphocyte Count (1)	CD8^+^ HLA-DR^+^Ki-67^+^T cells (1)	NA	CD4^+^ T cell (1), T_FH_(PD1^hi^CXCR5^hi^), T_eff_ (PD1^int^CXCR5^int^ or PD1^lo^CXCR5^lo^) (1)	CD8 T cell (1)
4. Blood immune biomarkers	NA	IL-18, ITAC, IFN-γ, IL-6 (1)	NA	NA	NA
5. Others	NA	Smoke history(3)	NA	FLIPIP1, FLIPI2(1)	NA

Note: TIL = tumor infiltrating lymophocyte; T_regs_ = regulatory T cell; T_FH_ = follicular helper T cell, T_eff_ = effector T cell, FLIPI = Follicular lymphoma international prognostic index; (*n*) = number of trials tested the biomarkers.

Meta-analysis showed that PD-L1 positive patients had a significantly higher response rate than PD-L1 negative patients (20 studies, RR = 1.92, 95% CI: 1.53–2.41, *P* < 0.001) with mild heterogeneity (*I*^2^ = 56.9%, χ^2^ = 44.08, *P* = 0.001) (Table 3 and Supplementary Figure S3A). Begg's test showed no evidence of substantial publication bias (*P* = 0.230). Compared to 265 out of 1000 people having response events in the PD-1 negative patients, 509 out of 1000 people had response cases in the PD-1 positive group. Based on a rate of 26.5% in the PD-1 negative group, the NNTB would be 4. Compared to PD-1 negative patients, the number of response cases added per 1000 individuals by PD-1 positive patients was 243. Subgroup analysis identified that PD-L1 positive patients had a significantly increased response rate during the treatment of all three anti-PD-1/PD-L1 antibodies Nivolumab (RR = 1.70, 95% CI: 1.32–2.17, *P* < 0.001), Pembrolizumab (RR = 2.56, 95% CI: 1.23–5.35, *P* < 0.001) and MPDL3280A (RR = 2.40, 95% CI: 1.48–3.88, *P* = 0.001) (Table 2 and Supplementary Figure S3B). Subgroup analysis also identified that PD-L1 positive melanoma (RR = 1.42, 95% CI: 1.22–1.65, *P* < 0.001), NSCLC (RR = 2.61, 95% CI: 1.87–3.65, *P* < 0.001) and RCC patients (RR = 1.91, 95% CI: 1.06–3.44, *P* = 0.032) had a significant increase in the response rates (Table 3 and Supplementary Figure S3C). Smoker patients also showed a significantly higher response rate than non-smoker patients (2 studies, RR = 5.45, 95% CI: 1.13–26.18, *P* = 0.034) without heterogeneity (*I*^2^ = 0.00%, χ^2^ = 0.22, *P* = 0.638) (Table 3 and Supplementary Figure S4A). However, there was no significant difference between BRAF mutation and previous Ipilimumab treatment history (Table 3, Supplementary Figure S4B and S4C). Due to the limited number of available publications, other factors weren't included in this meta-analysis.

**Table 3:** Meta-analysis of clinical responses based on PD-L1 expression, smoke history, BRAF and Ipilimumab treatment history

		Sample size		RR (95% CI)	*P* value	Study (*n*)
PD-L1 expression		Positive	Negative			
	Total	356/859 (41.4%)	411/1549 (26.5%)	1.92 (1.52, 2.41)	< 0.001	20
Subgroup (drug)	Nivolumab	231/547 (42.2%)	317/1034 (29.7%)	1.69 (1.32, 2.17)	< 0.001	14
	Pembrolizumab	87/187 (46.5%)	73/362 (20.2%)	2.56 (1.23, 5.35)	0.012	3
	MPDL3280A	38/125 (30.4%)	21/153 (13.7%)	2.39(1.48, 3.88)	< 0.001	3
Subgroup (tumor type)	Melanoma	190/340 (55.9%)	309/732 (42.2%)	1.42 (1.22, 1.65)	< 0.001	6
	NSCLC	100/306 (32.7%)	63/510 (12.4%)	2.61 (1.87, 3.65)	< 0.001	5
	RCC	19/86 (22.1%)	19/140 (13.6%)	1.91 (1.06, 3.44)	0.032	3
	Ovarian cancer	2/16 (12.5%)	1/4 (25.0%)	0·50 (0.06, 4.23)	0.525	1
	Bladder cancer	13/30 (43.35%)	4/35 (11.4%)	3.79 (1.38, 10.40)	0.010	1
	Mixed cancer*	32/87 (36.8%)	15/128 (11.7%)	4.61 (1.35, 15.74)	0.015	4
Smoke history (NSCLC)		Smoked	No-smoked			
	Total	31/92 (33.7%)	1/24 (4.2%)	6.02 (1.22, 29.75)	0.028	2
BRAF (Melanoma)		Wild	Mutation			
	Total	113/297 (38.0%)	23/75 (30.7%)	1.32 (0.92, 1.89)	0.128	3
Ipilimumab treatment (Melanoma)		Naive	Treated			
	Total	34/114 (29.8%)	26/93 (28.0%)	1.01 (0.64, 1.59)	0.969	2

* Mix cancer: multiple tumors included in these four studies but can't be separately totally by tumor types.

### Overall adverse effect rates

The overall pooled adverse effect rate of anti-PD-1/PD-L1 antibodies was 16% (95% CI: 12%–21%), (Table 1 and Supplementary Figure S5A). Egger's regression asymmetry test (*P* = 0.922) showed no evidence of substantial publication bias. Subgroup analyses also pooled the adverse effect rates for each drug and tumor (Table 1, Supplementary Figure S5B and S5C). The pooled adverse effect rates for Nivolumab and Pembrolizumab were 18% (95% CI: 11%–26%, Z = 7.70, *P* < 0.001) and 9% (95% CI: 6%–14%, Z = 7.43, *P* < 0.001), respectively. Anti-PD-1/PD-L1 antibodies combined with other therapies had a higher adverse effect rate of 29% (95% CI: 14%–46%, Z = 5.64, *P* < 0.001) than that of 13% with anti-PD-1/PD-L1 antibodies alone (95% CI: 10%–16%, Z = 14.98, *P* < 0.001) (Supplementary Figure S5D).

### Anti-PD-1 antibody showed higher safety compared with regular chemotherapy and Ipilimumab

**Figure 3:**
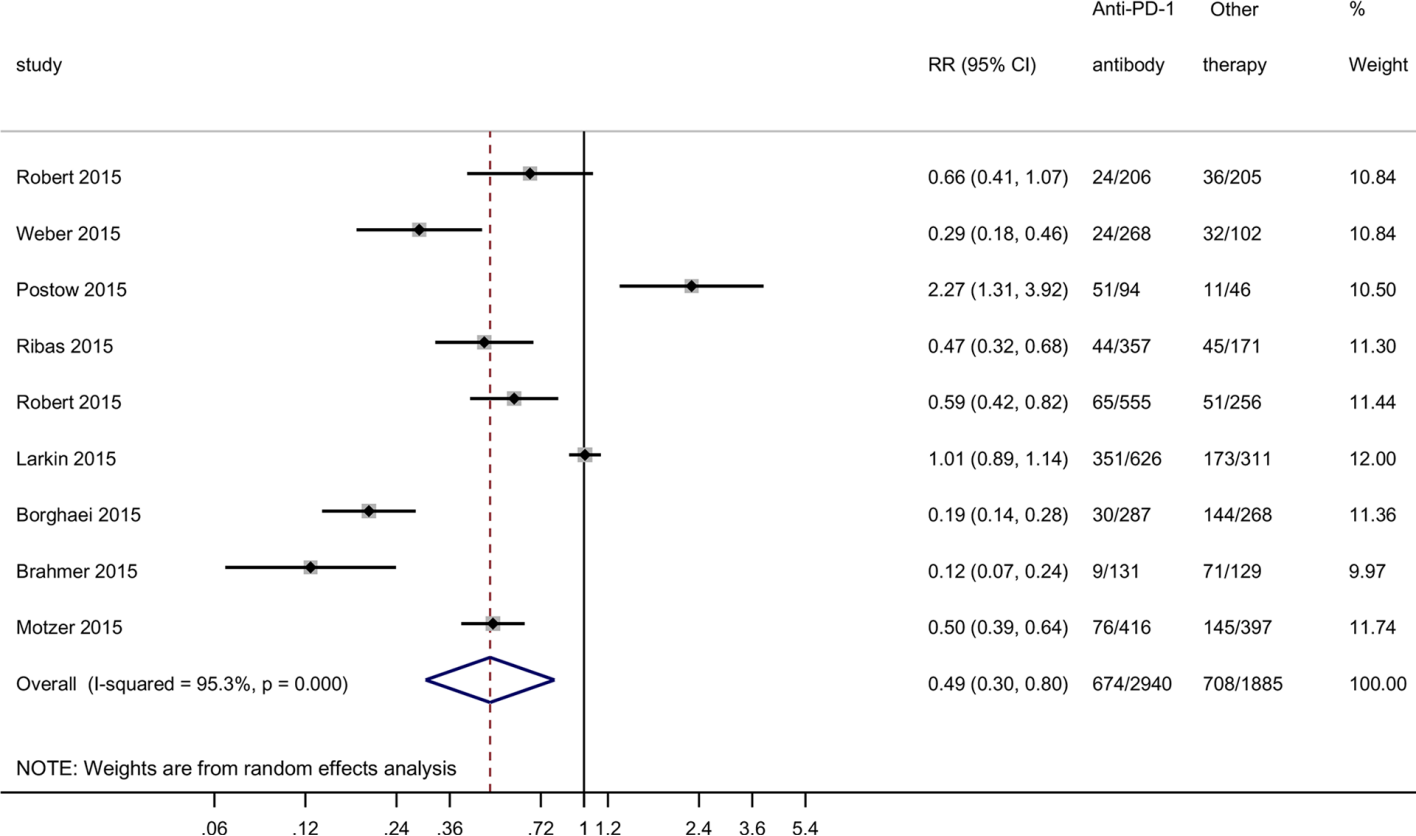
Forest plot for ratio risk and confidence intervals of adverse effect rate of anti-PD-1 antibody treatment compared with other therapies

We also compared the adverse effect rates of Nivolumab and Pembrolizumab with other therapies including chemotherapy and anti-CTLA4 antibody (clinical data showed in Supplementary Table S1). Meta-analysis showed that the anti-PD-1 antibody was associated with a significant decrease in adverse effect rate compared with other therapies (RR = 0.49, 95% CI: 0.30–0.80, *P* = 0.004), with large heterogeneity (*I*^2^ = 95.3, χ^2^ = 171.02, *P* < 0.001) (Figure 3). Egger's regression asymmetry test (*P* = 0.415) and Begg's test (*P* = 0.754) showed no evidence of substantial publication bias. Compared to 376 out of 1000 patients having adverse events in the group with other therapies, only 184 out of 1000 had adverse events with the anti-PD-1 antibody. Based on a rate of 37.6% in the group with other therapies, the NNTB would be 5. Compared to the group with other therapies, the number of adverse cases was reduced by 192 per 1000 individuals with the PD-1 antibody. Meta-regression analysis showed that Nivolumab/Ipilimumab combination was significantly positively associated with adverse effect rate (*P*= 0.022, Supplementary Table S3). Subgroup analysis also showed that Nivolumab/Ipilimumab combination increased adverse effects (RR = 1.45, 95% CI: 0.65–3.24), but did not reach statistical significance.

## DISCUSSION

In this study, meta-analysis of existing clinical studies demonstrated the high efficacy and safety of anti-PD-1/PD-L1 antibodies for various cancers, especially melanoma, NSCLC and RCC. The overall response rates of anti-PD-1/PD-L1 antibodies for advanced melanoma patients was 29%, which was higher than the 5–20% response rates from well-tolerated chemotherapies [23]. Ipilimumab, the first immune check point inhibitor targeted CTLA4, showed an objective response only 11% [24, 25]. We compared Nivolumab and Pembrolizumab with regular chemotherapy (including Ipilimumab) in melanoma patients. Nivolumab and Pembrolizumab showed higher response rate and reduced risk of death. The overall response rates of anti-PD-1/PD-L1 antibodies for NSCLC and RCC were both 21%. However, we didn't compare the anti-PD-1/PD-L1 antibodies with other therapies in NSCLC and RCC because there were only two studies that compared Nivolumab with Docetaxel in advanced NSCLC [26, 27] and only one study that compared Nivolumab with Everolimus in advanced RCC [28]. But in these trials, Nivolumab was reported with improved overall survival and response rates, as well as reduced adverse effect rates compared with regular chemotherapy. For other solid tumors, the response rates were limited due to the limited publications.

Our results showed a higher response rate in hematologic malignancy patients than solid tumor patients. Hematologic malignancies patients may more easily benefit from immunotherapy compared with solid tumors because of the insufficient T cell infiltration and highly immunosuppressive microenvironment in solid tumors [29]. In addition, some hematologic malignancies patients received anti-PD-1 antibody treatment after autologous stem-cell transplantation. This previous treatment might improve the immunological responses of anti-PD-1 antibody by restoring the body's ability to make normal blood cells.

PD-L1 expression might serve as a biomarker for anti-PD-1/PD-L1 antibody response. In our meta-analysis of pooled outcome from 20 trials, anti-PD-1/PD-L1 antibodies had the higher response rates in PD-L1 positive patients than that in PD-L1 negative patients. PD-L1 positive seems to predict the response for melanoma, NSCLC, RCC and bladder cancer patients. Concerning drugs, PD-L1 positive seems to predict the response for Nivolumab, Pembrolizumab and MPDL3280A. A similar result was reported by another group [30]. All of the PD-L1 expression results came from IHC staining. In most studies, IHC staining of more than 5% was defined as PD-L1 positive, but 10% and 1% were also used as the cut-off criterion in some studies. IHC staining platform, antibodies used and result evaluation method all contributed to the result heterogeneity. T1PD-L1 expression can be activated by monogenic signaling pathway PTEN loss, JAK/STAT, EGFR or cytokines released by immunologic response [32]. Since the PD-L1 expression is dynamic, the statuses of the tumor samples for detection, before, during or after previous treatment or immunotherapies also affected the PD-L1 IHC results [32, 33]. The distinct methods and interpretation in PD-L1 IHC assessment challenged the clinical application of PD-L1 expression as the biomarker for PD-1/PD-L1 antibodies.

Genetic mutation was an important factor associated with the responses of anti-PD-1/PD-L1 antibodies [34, 35]. Higher nonsynonymous mutation burden in tumors was associated with improved objective response, durable clinical benefit, and progression-free survival after anti-PD-1/PD-L1 antibody therapy. For instance, mismatch-repair status (DNA repair and replication gene mutations such as POLD1, POLE and MSH2) could predict clinical benefit of Pembrolizumab for colon cancer patients and the high somatic mutation burden was associated with prolonged PFS survival [16]. In NSCLC, KRAS mutation positive patients had better overall survival from Nivolumab than docetaxel [29]. In Gettinger's study, responses were seen in patients with EGFR/KRAS wild type and EGFR/ KRAS mutant received Nivolumab [11].

Genetic mutation and epigenetic modification could alter the expression of tumor associated self-antigens, induce more neoantigens and enhance tumor antigenicities, thereby increasing the anti-tumor immune responses in tumor microenvironments [36]. Neoantigen was identified as the tumor-specific antigen arisen as a consequence of tumor-specific mutations. Recent research showed that recognition of patient-specific neoantigens is a major factor in the activity of clinical anti-PD-1/PD-L1 immunotherapy [36]. It's possible that the burden of candidate neoantigens correlated with anti-PD-1/PD-L1 antibody response, but not the frequency per nonsynonymous mutation. s with presenting in approximately 40% of melanoma patients37, 8Ourm Areported a similar result: after ing9This may be due to the limited population, but could also be because a single BRAF V600 mutation is not enough to work as the biomarker for anti-PD-1/PD-L1 antibodies.

Meanwhile, ometa-analysis a higher response rate inSve4 Therefore smoker patients may have a higher burden of candidate neoantigens, which was correlated with higher response rate for PD-1/PD-L1 antibodies. eda These results suggested that perhaps a systematic evaluation on tumor neoantigen landscape but not a single specific factor should be employed to predict the response for anti-PD-1/PD-L1 antibody.

Immune checkpoint inhibitors have also been linked to the development of certain adverse events. The comment treatment related adverse effects have rash, diarrhea, fatigue, neutropenia, decreased platelet count, thrombocytopenia, hypothyroidism and others [8–11]. The overall adverse effect rate pooled in our meta-analysis results was 16%. Anti-PD-1/PD-L1 antibodies are well tolerated in patients. Similar adverse effects were reported in Pembrolizumab and Nivolumab [8, 40, 41]. The safety profiles of different anti-PD-1/PD-L1 antibodies or tumors are similar except for Pidilizumab in the treatment of hematologic malignancies. This might be because most patients recruited in these two Pidilizumab trials were patients with hematologic malignancies. The subgroup analysis showed higher adverse effect rate in patients with hematologic malignancies (30%) compared with solid tumors (9–26%). Even so, the adverse effect rate of anti-PD-1/PD-L1 antibodies for patients with hematologic malignancies was still lower than that of other regular therapies, even in contrast to anti-CTL4 antibody. Moreover, our result showed Nivolumab and Pembrolizumab could reduce adverse effects compared with other therapies. Concerning the control type, our data analysis didn't show a lower adverse effect rate of Nivolumab and Pembrolizumab when compare with Ipilimumab. This may be due to the limited trial included in the meta-analysis. Interestingly, our results found that combination with other therapies has the potential to increase the adverse effect rate as well as to increase the response rate. Blocking of PD-1 alone might not fully restore the function of antitumor T cells because of other inhibitory receptors such as CTL-4, LAG3, TIM3, BTLA, CD160, and CD244 [5, 14, 42]. Our findings suggest the necessity of to balancing the clinical outcome with the adverse effects when using combination strategies of anti-PD-1/PD-L1 antibodies for advanced cancer patients.

Our review also has limitations. Firstly, high heterogeneity was found when we pooled the overall response rate and adverse effect rate. Because of participated heterogenerity, we used random-effects model to pool studies and performed subgroup analysis and meta-regression analysis. NSCLC, combination therapy and antigen origin positively associated with anti-PD-1/PD-L1 antibody responses. The heterogeneity was reduced in NSCLC and anti-PD-L1 antibody and some other subgroups, but high heterogeneity was still found in most part of subgroups. Since no evidence of substantial publication bias was found, these results suggested the necessity of valuable biomarkers to evaluate clinical outcomes of anti-PD1/PD-L1 antibodies. Secondly, few trials were collected for some outcomes such as comparing the response rate and adverse effects rate between anti-PD-1/PD-L1 antibodies with other therapy, and comparing response rate between smoke history, BRAF mutation status, and previous Ipilimumab treatment. More research is needed to understand and evaluate these clinical outcomes.

In conclusion, our meta-analysis with existing clinical studies verified the high efficacy and safety of anti-PD-1/PD-L1 antibodies in melanoma, NSCLC, RCC and other cancers. More importantly, the results of this meta-analysis supported the notion that PD-L1 expression and smoke history are correlated with better clinical responses of anti-PD-1/PD-L1 antibodies. Our study is the most up-to-date updated meta-analysis to present an evaluation for efficacy and safety of anti-PD-1/PD-L1 antibodies for the treatment of advanced or refractory cancer patients, which supports future clinical applications for anti-PD-1/PD-L1 antibody-based immunotherapy.

## MATERIALS AND METHODS

### Data sources and searches

The deadline for trial publication and/or presentation was January 31th, 2016. Systematic literature searches were conducted in PubMed and EMBASE according to Cochrane guidelines [43]. Search key terms included PD-1, PD-L1, Nivolumab, Pembrolizumab, Lambrolizumab, MPDL3280A, BMS-936559, cancer, tumor, carcinoma, phase I, phase II, phase III. We also manually searched references in identified studies in case of missing trials.

### Study selection

The eligible studies were limited to human clinical trials published in English. Studies were included if they performed clinical trials with anti-PD-1/PD-L1 antibodies for advanced or refractory cancer patients, reporting either the efficiency or safety. Meeting abstracts in the EMBA database from ASCO (www.asco.org) and ESMO (www.esmo.org) published in the last two years were also included. When part or all of the patients were involved in more than one publication, only the most complete or most informative study was included in this analysis.

### Data extraction and quality assessment

All the eligible papers were reviewed and extracted independently by two investigators, and the disagreements were resolved by discussion; a third investigator adjudicated the controversial parts. For each included study, data set was extracted from each article by patients' tumor type. Extracted data included followings: adopted drugs, tumor types, sample size, patients' previous treatment status, combination strategies, control setup, patients' response, PFS, median PFS data, Hazard ratios (HRs), Grade 3 and 4 adverse effects, and potential biomarkers to predict which patients might benefit from anti-PD-1/PD-L1 antibodies if assessed in the manuscript.

The primary endpoint was patients' response to anti-PD-1/PD-L1 antibodies. The responses were derived from independent, central, blinded radiologic review, with assessment according to the Response Evaluation Criteria in Solid Tumors (RECIST) [44]. Patients with responses to anti-PD-1/PD-L1 antibody immunotherapy were divided into two groups: positive response group [patients achieved complete response (CR, all target lesions disappearance) or partial response (PR, at least a 30% decrease in the sum of the longest diameter of target lesions)], and negative response group [patients achieved stable disease (SD, neither sufficient shrinkage to qualify for PR nor sufficient increase to qualify for PD), progress disease (PD, at least a 20% increase in the sum of the longest diameter of target lesions)]. The overall response rates were the percentage of patients that achieved complete response and partial response. The second endpoint included the safety and survival. Grade 3 and 4 adverse effects were graded according to the National Cancer Institute Common Toxicity Criteria. Survival was indicated by progression-free survival (PFS).

In order to ensure the quality of the meta-analysis, two authors independently evaluated the quality of the studies included in the systematic review using the Methodological Index for Nonrandomized Studies (MINORS) [45]. The MINORS scale included 8 criteria for nonrandomized studies and additional 4 criteria in the case of comparative studies.

### Data synthesis and analysis

For clinical response and adverse effect rate, the overall rate was pooled by Metaprop in Stata. Metaprop is a statistical program implemented to perform meta-analyses of proportions as mentioned in our former study [46, 47]. It provides appropriate methods for dealing with proportions close to 0% or 100%. Cochran's *Q* test was used to assess between-study differences and the I^2^ statistic to quantify the proportion of observed inconsistency across study results not explained by chance. If the heterogeneity among trials was very large (*I*^2^ statistic > 75%), a random effects meta-analysis was used. The pooled response rates or adverse effect rates describe the mean of the distribution of the estimated response rates. To compare response rates and adverse effect rates of anti-PD-1/PD-L1 antibodies with other therapy regimens and the response rates between different biomarker statuses, the relative ratio (RR) was calculated. To analyze the PFS, the hazard ratios (HRs) and their CIs were estimated using the methods proposed by Parmer [48]. When there was an effect, a number needed to treat for an additional beneficial outcome (NNTB) or number needed to treat for an additional harmful outcome (NNTH) was calculated from the RR.

To explore the sources of heterogeneity, meta-regression analyses were conducted by pre-defined subgroups including adopted drugs, tumor types, combination strategies and antigen origin. We also compared pooled results obtained from subsets of studies grouped according to the tumor type, drugs used and combination strategies. Univariate meta-regression analyses were conducted to identify clinical factors associated with adverse effects. Next, we performed a multivariable meta-regression analysis including the significant factors in the univariate analysis. Potential interaction was also tested between potential predictors. Publication bias was analyzed by both Begg's and Egger's regression asymmetry test, and visually evaluated using the funnel plot. Stata Statistical Software (version 13.0 Stata Corp., College Station, TX, USA) was used for all analyses. A two-sided *P* value ≤ 0.05 was considered as statistically significant.

## SUPPLEMENTARY MATERIALS FIGURES AND TABLES




