# Supplementary material for: The efficacy and safety of anti-PD-1/PD-L1 antibodies for treatment of advanced or refractory cancers: a meta-analysis
Source: Oncotarget. 2016 Sep 24;7(45):73068–79. doi: 10.18632/oncotarget.12230 (PMC5341964; doi:10.18632/oncotarget.12230)
Supplement: Supplementary file 1 [file oncotarget-07-73068-s001.pdf]

The efficacy and safety of anti-PD-1/PD-L1 antibodies for treatment of advanced or refractory cancers: a meta-analysis

Supplementary Materials

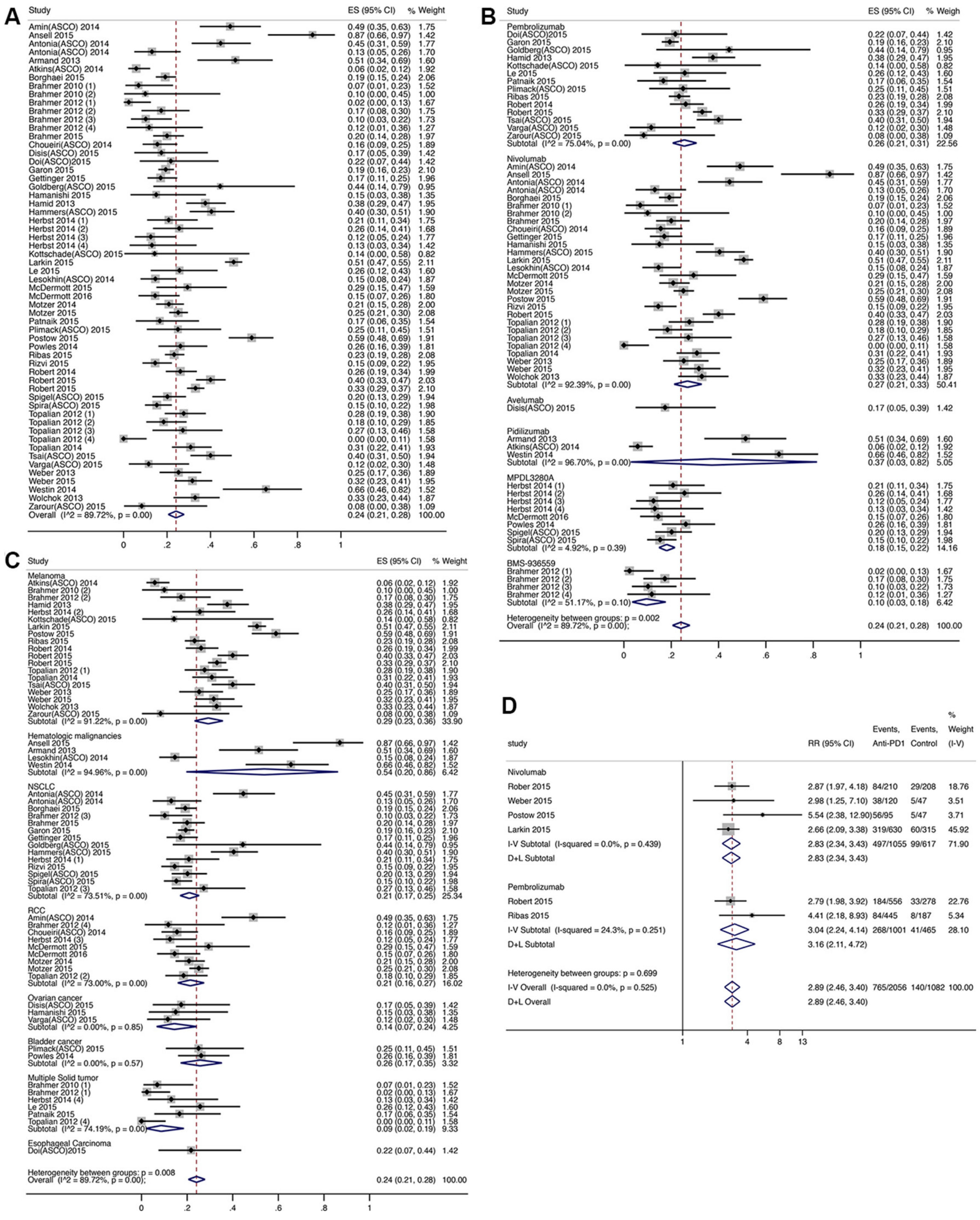

Supplementary Figure S1: Meta analysis of anti-PD-1/PD-L1antibody response rates. (**A**) Forest plot for ratio risk and confidence intervals of anit-PD-1/PD-L1 antibody overall response rates. (**B**) Forest plot for ratio risk and confidence intervals of subgroup analysis of anit-PD-1/PD-L1 antibody response rates divided by different drugs. (**C**) Forest plot for ratio risk and confidence intervals of anit-PD-1/PD-L1 antibody response rates divided by different tumor types. (**D**) Forest plot for ratio risk and confidence intervals of anit-PD-1/PD-L1 antibody response rates compared with other therapies divided by different drugs.

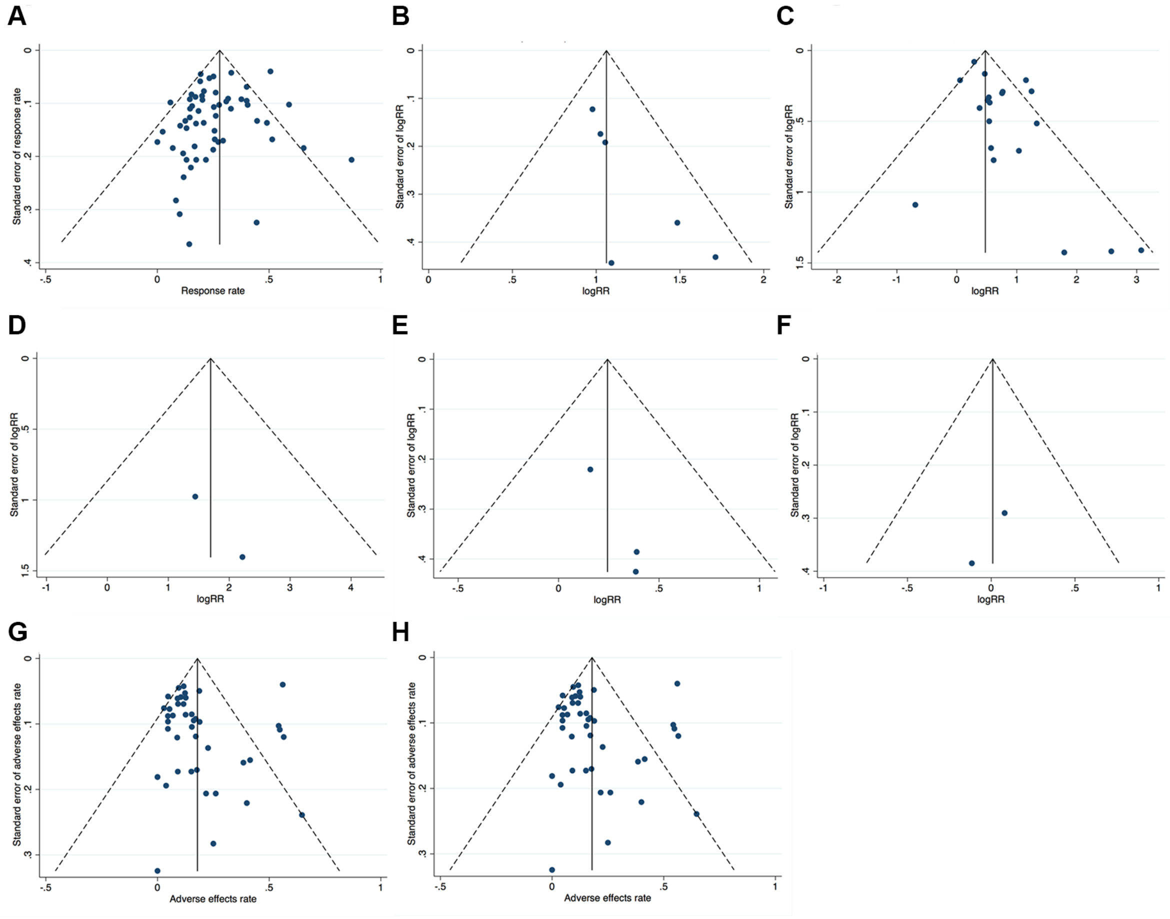

Supplementary Figure S2: Funnel plot of each Meta-analysis. (**A**) Overall response rates of anti-PD-1/PD-L1antibodies, (**B**) Response rates of anti-PD-1/PD-L1 antibodies compared with other therapies, (**C**) Response rates of PD-L1 positive patients compared with PD-L1 negative patients, (**D**) Response rates of smoked patients compared with non-smoked patients, (**E**) Response rates of BRAF wildtype patients compared with BRAF mutated patients, (**F**) Response rates of Ipilimumab naïve patients compared with Ipilimumab treated patients, (**G**) Overall adverse effect rate of anti-PD-1/PD-L1 antibodies, (**H**) Adverse effect rates of anti-PD-1/PD-L1 antibodies compared with other therapies.

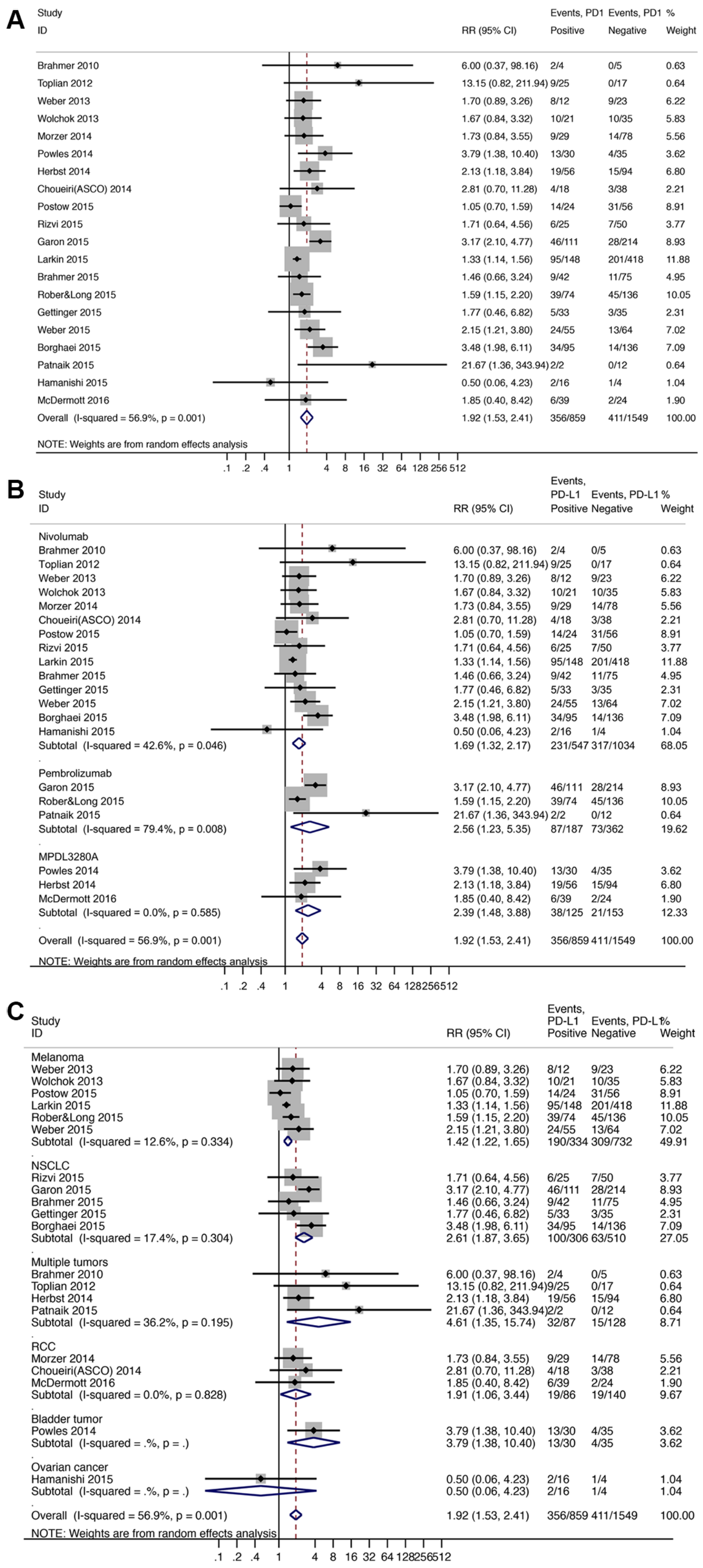

Supplementary Figure S3: Meta-analysis of PD-L1 expression to predict anti-PD-1/PD-L1 antibody response rates. (**A**) Forest plot for ratio risk and confidence intervals of response rates in PD-L1 positive patients compared with PD-L1 negative patients. (**B**) Forest plot for subgroup analysis of response rates in PD-L1 positive patients compared with PD-L1 negative patients divided by different drugs. (**C**) Forest plot for subgroup analysis of response rates in PD-L1 positive patients compared with PD-L1 negative patients divided by different tumor types.

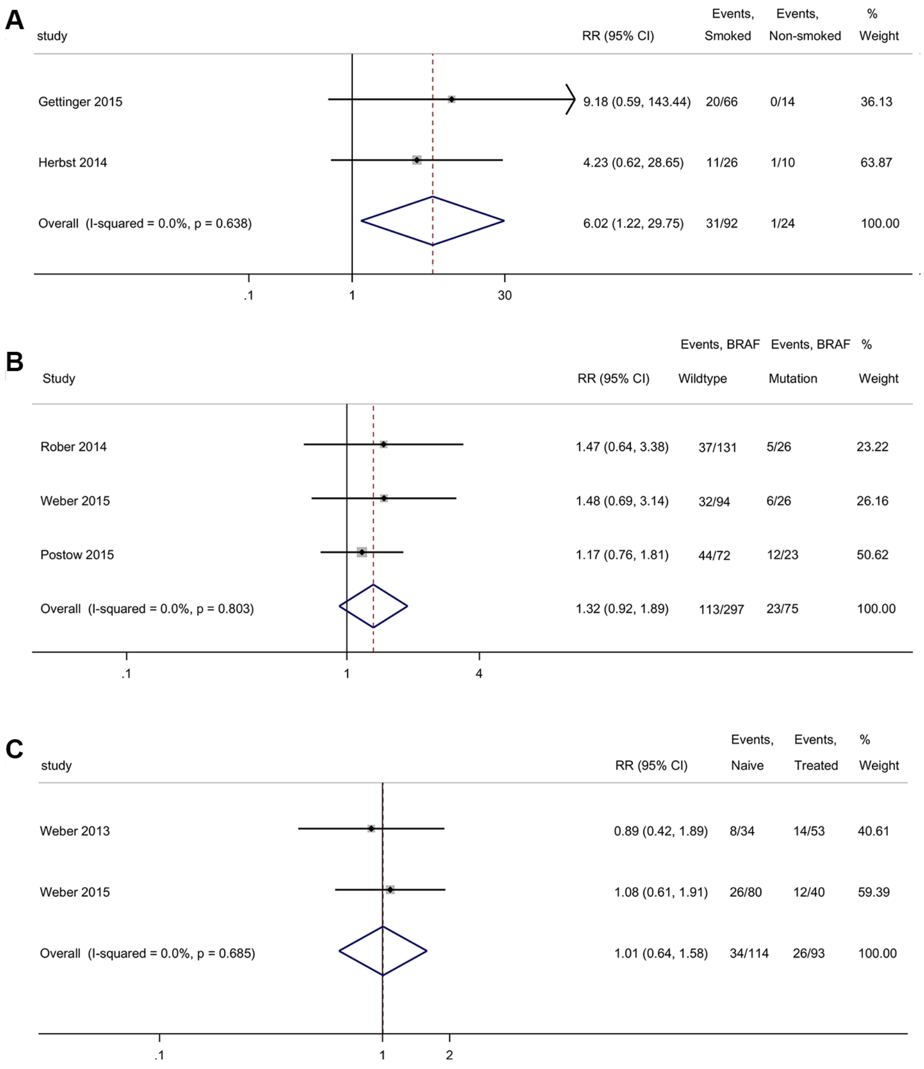

Supplementary Figure S4: Meta-analysis of other potential biomarkers to predict anti-PD-1/PD-L1 antibody response rates. (**A**) Forest plot for ratio risk and confidence intervals of response rates in smoked patients compared with non-smoked patients. (**B**) Forest plot for ratio risk and confidence intervals of response rates in BRAF wildtype patients compared with BRAF mutated patients. (**C**) Forest plot for ratio risk and confidence intervals of response rates in Ipilimumab naïve patients compared with Ipilimumab treated patients.

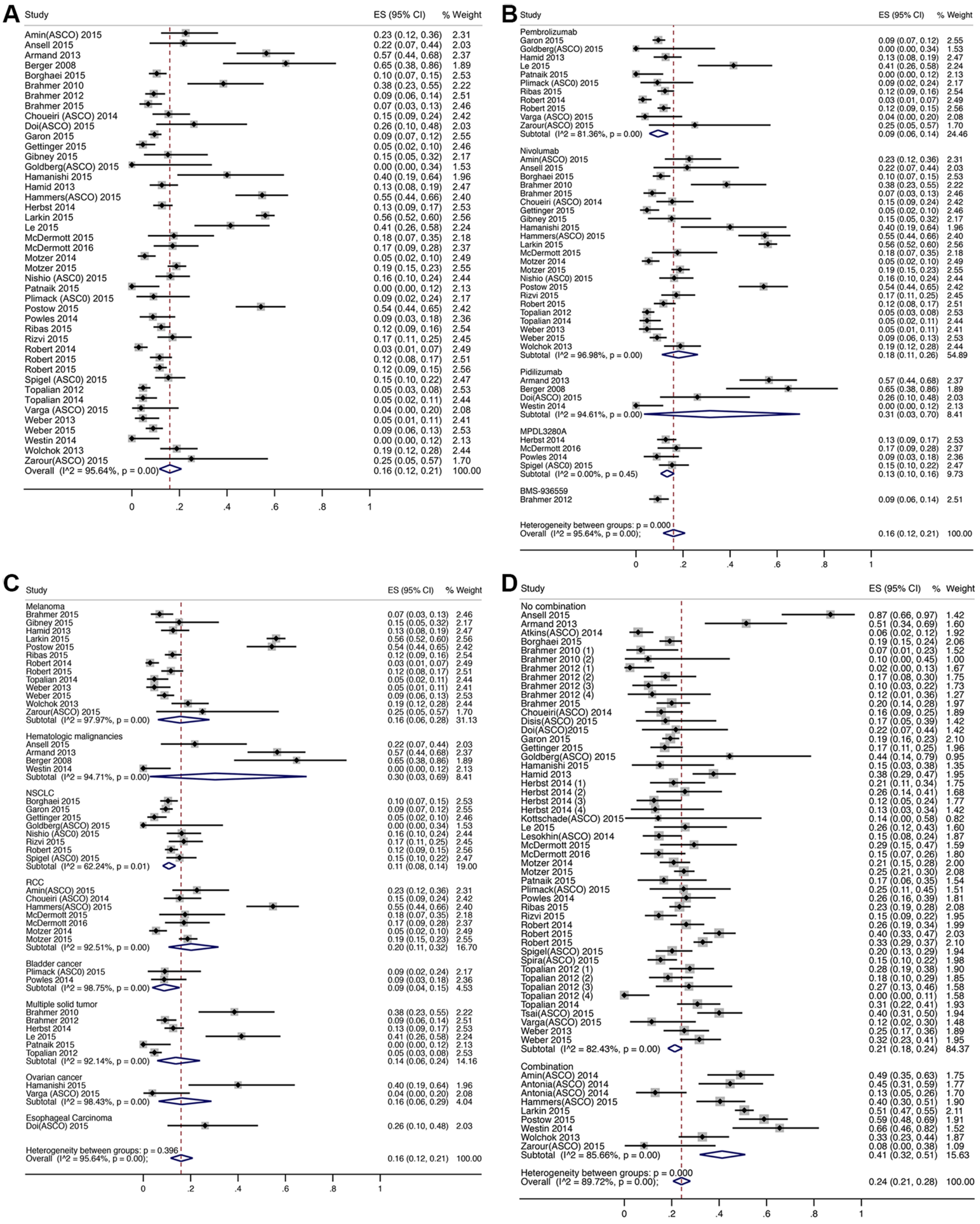

Supplementary Figure S5: Meta-analysis of anti-PD-1/PD-L1 antibody adverse effects rates. (**A**) Forest plot for ratio risk and confidence intervals of anit-PD1/PD-L1 antibody overall adverse effect rates. (**B**) Forest plot for ratio risk and confidence intervals of anit-PD1/PD-L1 antibody adverse effect rates divided by different drugs. (**C**) Forest plot for ratio risk and confidence intervals of anit-PD1/PD-L1 antibody adverse effect rates divided by different tumor types. (**D**) Forest plot for ratio risk and confidence intervals of anit-PD1/PD-L1antibody adverse effect rates divided by combination strategy.

Supplementary Table S1: Summary of clinical trials with anti-PD-1/PD-L1 antibodies involved in the meta-analysis. See Supplementary_Table_S1

Supplementary Table S2: Quality assessment

Study	Non-randomized studies								Additional criteria in comparative study				Total
	A	B	C	D	E	F	G	H	I	J	K	L	
Berger 2008	2	2	2	2	2	2	2	0					14
Brahmer 2010	2	2	2	2	0	0	0	0					8
Topalian 2012	2	2	2	2	0	2	1	2					13
Brahmer 2012	2	2	2	2	0	0	0	2					10
Hamid 2013	2	2	2	2	0	2	1	2					13
Weber 2013	2	2	2	2	0	2	2	2					14
Wolchok 2013	2	2	2	2	0	2	2	2					14
Armand 2013	2	2	2	2	0	2	1	2					13
Topalian 2014	2	2	2	2	0	2	2	0					12
Powles 2014	2	2	2	2	0	2	2	0					12
Westin 2014	2	2	2	2	0	2	2	2					14
Herbst 2014	2	2	2	2	0	2	2	0					12
Ansell 2015	2	2	2	2	0	2	2	2					14
Garon 2015	2	1	2	2	0	2	1	2					12
Gibney 2015	2	2	2	2	0	2	2	0					12
Le 2015	2	2	2	2	0	2	2	2					14
McDermott 2015	2	2	2	2	0	2	2	0					12
Gettinger 2015	2	2	2	2	0	2	2	0					12
Rizvi 2015	2	2	2	2	0	2	2	2					14
McDermott 2016	2	2	2	2	0	2	2	0					12
Weber 2015	2	2	2	2	0	2	0	2	2	2	2	2	20
Larkin 2015	2	2	2	2	2	2	2	2	1	2	2	2	23
Brahmer 2015	2	2	2	2	0	2	2	2	2	2	2	2	22
Robert L 2015	2	2	2	2	0	2	2	2	2	2	2	2	22
Robert 2014	2	2	2	2	0	2	2	2	1	2	2	2	21
Motzer 2014	2	2	2	2	0	2	2	2	1	2	2	2	21
Ribas 2015	2	2	2	2	0	2	2	2	2	2	2	2	22
Robert S 2015	2	2	2	2	0	2	2	2	1	2	2	2	21
Postow 2015	2	1	2	2	0	2	2	2	2	2	2	2	21
Motzer 2015	2	2	2	2	0	2	1	0	2	2	2	2	19
Borghaei 2015	2	2	2	2	0	2	1	0	2	2	2	2	19

A = Clear aim; B = Inclusion of consecutive patients; C = Prospective collection of data; D = Appropriate Endpoint; E = Unbiased assessment; F = Follow-up period; G = Loss to follow up less than 5%; H = Prospective calculation of study size; I = Adequate control group; J = Contemporary groups; K = Baseline equivalence; L = Adequate statistical analyses.

Supplementary Table S3: Meta-regression analysis results for heterogeneity with adverse effects when anti-PD-l antibodies compared with other therapies

	RR		Meta-regression	
	**RR (95% CI)**	***P***	**Coefficients (95% CI)**	***P***
Drug				
Niovlumab	0.48 (0.25, 0.90)	0.023	–0.10 (–1.81, 1.62)	0.889
Pembrolizumab	0.53 (0.41, 0.68)	< 0.001		
Combination strategy				
Combined with Ipilimumab	1.45 (0.65, 3.24)	0.369	1.39 (0.27, 2.52)	0.022
No Combination	0.36 (0.24, 0.54)	< 0.001		
Tumors				
Melanoma	0.70 (0.44, 1.11)	0.127	0.33 (–1.31, 1.98)	0.638
NSCLC	0.17 (0.11, 0.25)	< 0.001	–1.15 (–3.05, 0.76)	0.191
RCC	0.50 (0.39, 0.80)	< 0.001	NA	NA
Control setup				
Chemotherapy	0.33 (0.21, 0.52)	< 0.001	–1.18 (–2.23, –0.13)	0.032
Ipilimumab	1.05 (0.61, 1.81)	0.853		
