# Supplementary material for: The efficacy and safety of anti-PD-1/PD-L1 antibodies for treatment of advanced or refractory cancers: a meta-analysis
Source: Oncotarget. 2016 Sep 24;7(45):73068–79. doi: 10.18632/oncotarget.12230 (PMC5341964; doi:10.18632/oncotarget.12230)
Supplement: Supplementary file 2 [file oncotarget-07-73068-s002.docx]

Supplementary Table S1 Summary of clinical trials with anti-PD-1/PD-L1 antibodies involved in the meta-analysis

| Study | Drug | Phase ^a^ | Sample size ^b^ | Efficacy ^c^ | Adverse effects ^d^ | Cancers | Patients tumor status | Combination | Control | Ref |
| --- | --- | --- | --- | --- | --- | --- | --- | --- | --- | --- |
| Hamid, 2013 | Pembrolizumab | I | 135 |  | 135 | Melanoma | Advanced, received prior treatment with ipilimumab or not |  |  | 1 |
| Weber, 2013 | Nivolumab | I | 90 | 87 | 90 | Melanoma | unresectable stage III or IV melanoma, progressive disease after at least one previous systemic treatment |  |  | 2 |
| Wolchok, 2013 | Nivolumab | I | 86 | 82 | 86 | Melanoma | unresectable, stage III or IV melanoma, received previous treatment | Ipilimumab |  | 3 |
| Atkins(ASCO), 2014 | Pidilizumab | II | 103 | 103 |  | Melanoma | Stage IV clearly progressive; ECOG 0-1; prior systemic therapies |  |  | 4 |
| Robert, 2014 | Pembrolizumab | I | 173 | 157 | 173 | Melanoma | ipilimumab-refractory advanced melanoma |  |  | 5 |
| Topalian, 2014 | Nivolumab | I/II | 107 | 107 | 107 | Melanoma | advanced stage, received therapy |  |  | 6 |
| Gibney, 2015 | nivolumab | I | 33 |  | 33 | Melanoma | surgically resected stage IIIC or IV melanoma | Multi-peptide vaccine |  | 7 |
| Robert, 2015 | Nivolumab | III | 209 | 210 | 206 | Melanoma | previously untreated patients who had metastatic melanoma |  | Dacarbazine | 8 |
| Weber, 2015 | Nivolumab | III | 120 | 120 | 272 | Melanoma | advanced melanoma who progressed after anti-CTLA-4 treatment |  | ICC ^1^ | 9 |
| Postow, 2015 | Nivolumab | II | 95 | 95 | 94 | Melanoma | metastatic melanoma with not previously received treatment | Ipilimumab | Ipilimumab | 10 |
| Larkin, 2015 | Nivolumab | III | 630 | 630 | 626 | Melanoma | stage III (unresectable) or stage IV melanoma and had received no prior systemic treatment | Ipilimumab | Ipilimumab | 11 |
| Robert, 2015 | Pembrolizumab | III | 556 | 556 | 555 | Melanoma | 65.8% had received no previous systemic treatment |  | Ipilimumab | 12 |
| Ribas, 2015 | Pembrolizumab | II | 361 | NA | 356 | Melanoma | ipilimumab-refractory melanoma |  | ICC ^2^ | 13 |
| Kottschade(ASCO), 2015 | Pembrolizumab |  | 7 | 7 |  | Melanoma | unresectable metastatic melanoma, had progressed on prior ipilimumab or BRAF inhibitor therapy |  |  | 14 |
| Tsai(ASCO), 2015 | Pembrolizumab |  | 110 | 110 |  | Melanoma | Advanced melanoma |  |  | 15 |
| Zarour(ASCO), 2015 | Pembrolizumab |  | 12 | 12 | 12 | Melanoma | stage III and metastatic stage IV melanoma | Peg-IFN |  | 16 |
| Antonia(ASCO), 2014 | Nivolumab |  | 56 | 56 |  | NSCLC | advanced NSCLC | Chemptherapy |  | 17 |
| Antonia(ASCO), 2014 | Nivolumab | I | 46 | 46 |  | NSCLC | Chemotherapy-naive pts | Ipilimumab |  | 18 |
| Rizvi,2015 | Nivolumab | II | 117 | 117 | 117 | NSCLC | stage IIIB or IV, recurrence after previous treatment |  |  | 19 |
| Garon,2015 | Pembrolizumab | I | 495 | 495 | 495 | NSCLC | advanced or metastatic, progressed after previous treatment or naïve to treatment |  |  | 20 |
| Brahmer,2015 | Nivolumab | III | 272 | 135 | 131 | NSCLC | advanced, progression during or after first-line chemotherapy |  | Docetaxel | 21 |
| Gettinger,2015 | Nivolumab | I | 129 | 129 | 129 | NSCLC | heavily pretreated advanced NSCLC |  |  | 22 |
| Borghaei,2015 | Nivolumab | III | 287 | 30 | 287 | NSCLC | progressed during or after platinum-based doublet chemotherapy |  | Docetaxel | 23 |
| Spigel(ASCO), 2015 | MPDL3280A | II | 138 | 114 | 137 | NSCLC | chemo-naive pts or with treated asymptomatic brain metastases |  |  | 24 |
| Nishio(ASCO), 2015 | Nivolumab | II | 111 |  | 111 | NSCLC | previously treated advanced NSCLC |  |  | 25 |
| Spira(ASCO), 2015 | MPDL3280A | II | 144 | 144 |  | NSCLC | Previously treated NSCLC |  | Docetaxel | 26 |
| Goldberg(ASCO), 2015 | Pembrolizumab | II | 9 | 9 | 9 | NSCLC | have ≥1 Brain metastasis that is previously untreated or progressing after prior local therapy |  |  | 27 |
| Amin(ASCO), 2014 | Nivolumab | I | 53 | 53 | 53 | RCC | Metastatic RCC received more than one prior systemic treatment | Sunitinib or Pazopanib |  | 28 |
| Choueiri(ASCO), 2014 | Nivolumab |  | 91 | 90 | 91 | RCC | previously treated or untreated metastatic RCC |  |  | 29 |
| Motzer,2014 | Nivolumab | II | 168 | 168 | 167 | RCC | metastatic renal cell received prior treatment |  |  | 30 |
| McDermott,  2015 | Nivolumab | I | 34 | 34 | 34 | RCC | previously treated advanced RCC |  |  | 31 |
| Motzer,2015 | Nivolumab | III | 406 | 103 | 410 | RCC | received previous treatment with one or two regimens of antiangiogenic therapy |  | Everolimus | 32 |
| Hammers(ASCO), 2015 | Nivolumab | I | 94 | 94 | 94 | RCC | Metastatic RCC | Ipilimumab |  | 33 |
| McDermott,  2016 | MPDL3280A | Ia | 70 | 62 | 70 | RCC | Metastatic RCC |  |  | 34 |
| Powles,2014 | MPDL3280A | I | 68 | 65 | 68 | Bladder cancer | Metastatic UBC received previous treatment |  |  | 35 |
| Hamanishi,  2015 | Nivolumab |  | 20 | 20 | 20 | Ovarian Cancer | Advanced or relapsed platinum-resistant ovarian cancer |  |  | 36 |
| Disis(ASCO), 2015 | Avelumab | Ib | 23 | 23 |  | Ovarian cancer | previously treated, recurrent or refractory ovarian cancer |  |  | 37 |
| Varga(ASCO), 2015 | Pembrolizumab |  | 26 | 26 | 26 | Ovarian cancer |  |  |  | 38 |
| Berger,2008 | Pidilizumab | I | 17 | 17 | 17 | Hematologic malignancies | advanced stage, |  |  | 39 |
| Armand,2013 | pidilizumab | II | 66 | 35 | 69 | DLBCL | chemotherapy sensitive disease |  |  | 40 |
| Westin,2014 | pidilizumab | II | 32 | 29 | 30 | Follicular lymphoma | rituximab-sensitive follicular lymphoma relapsing after one to four previous therapies | Rituximab |  | 41 |
| Ansell,2015 | Nivolumab | I | 23 | 23 | 23 | Hodgkin’s Lymphoma | Relapsed or Refractory Hodgkin’s Lymphoma |  |  | 42 |
| Lesokhin(ASCO), 2014 | Nivolumab | I | 82 | 82 |  | lymphoid malignancies | relapsed or refractory lymphoid malignancies |  |  | 43 |
| Doi(ASCO), 2015 | Pembrolizumab | Ib | 23 | 23 | 23 | Esophageal carcinoma | Advanced solid tumor |  |  | 44 |
| Brahmer,2010 | Nivolumab | I | 39 | 39 | 39 | Mmelanoma, CRC, PrC, NSCLC,RCC | treatment-refractory metastatic melanoma |  |  | 45 |
| Brahmer,2012 | BMS-936559 | I | 207 | 160 | 2 | NSCLC, melanoma, CRC  RCC, OC, PC, GC, BC | advanced stage, received therapy |  |  | 46 |
| Topalian,2012 | Nivolumab | I | 296 | 203 | 296 | Melanoma, NSCLC, RCC,  PrC, CRC | advanced stage, received therapy |  |  | 47 |
| Herbst,2014 | MPDL3280A | Ia | 227 | 175 | 277 | NSCLC, melanoma, RCC, Others | incurable, or metastatic |  |  | 48 |
| Le,2015 | Pembrolizumab | II | 41 | 35 | 41 | Colon cancer and other solid tumors | treatment-refractory progressive  metastatic cancer |  |  | 49 |
| Patnaik 2015, | Pemrolizumab | I | 30 | 30 | 30 | melanoma, NSCLC and other slid tumors | advanced solid tumor, experienced disease progression on or intolerant of or not eligible for standard therapy |  |  | 50 |
| Plimack(ASCO), 2015 | Pembrolizumab |  | 33 | 28 | 33 | bladder, renal pelvis, ureter, or urethra cancer | recurrent, metastatic, or persistent urothelial cancer of the bladder, renal pelvis, ureter, or urethra |  |  | 51 |

Note: ^a^ clinical trial phase status; ^b^ total patient involved in trial; ^c^ total patients eligible for efficiency (clinical response) analysis; ^d:^ total patients eligible for grade 3 and 4 adverse effects analysis; CRC= colorectum cancer; PrC= prostate cancer; NSCLC= non small cell lung cancer; RCC= renal cell carcinoma; OC= ovarian cancer; PC=pancreatic cancer; GC=gastric cancer; BC=breast cancer; ICC ^1^ = dacarbazine 1000 mg/m² every 3 weeks or paclitaxel 175 mg/m² combined with carboplatin area under the curve 6 every 3 weeks, ICC^2^ = paclitaxel plus carboplatin, paclitaxel, carboplatin, dacarbazine, or oral temozolomide

Reference:

1. Hamid O, Robert C, Daud A, Hodi FS, Hwu WJ, Kefford R, et al. Safety and tumor responses with lambrolizumab (anti-PD-1) in melanoma. .N Engl J Med 2013;369:134-44.

2. Weber JS, Kudchadkar RR, Yu B, Gallenstein D, Horak CE, Inzunza HD, et al. Safety, efficacy, and biomarkers of nivolumab with vaccine in ipilimumab-refractory or -naive melanoma. J Clin Oncol 2013;31:4311-8.

3. Wolchok JD, Kluger H, Callahan MK, Postow MA, Rizvi NA, Lesokhin AM, et al. Nivolumab plus ipilimumab in advanced melanoma. N Engl J Med 2013;369:122-33.

4. Atkins MB, Kudchadkar RR, Sznol M, McDermott DF, Lotem M, Schachter J, et al. Phase 2, multicenter, safety and efficacy study of pidilizumab in patients with metastatic melanoma. J Clin Oncol. 2014;32.

5. Robert C, Ribas A, Wolchok JD, Hodi FS, Hamid O, Kefford R, et al. Anti-programmed-death-receptor-1 treatment with pembrolizumab in ipilimumab-refractory advanced melanoma: a randomised dose-comparison cohort of a phase 1 trial. Lancet. 2014;384:1109-17.

6. Topalian SL, Sznol M, McDermott DF, Kluger HM, Carvajal RD, Sharfman WH, et al. Survival, durable tumor remission, and long-term safety in patients with advanced melanoma receiving nivolumab. J Clin Oncol. 2014;32:1020-30.

7. Gibney GT, Kudchadkar RR, DeConti RC, Thebeau MS, Czupryn MP, Tetteh L, et al. Safety, correlative markers, and clinical results of adjuvant nivolumab in combination with vaccine in resected high-risk metastatic melanoma. Clin Cancer Res 2015;21:712-20.

8. Robert C, Long GV, Brady B, Dutriaux C, Maio M, Mortier L, et al. Nivolumab in previously untreated melanoma without BRAF mutation. N Engl J Med 2015;372:320-30.

9. Weber JS, D'Angelo SP, Minor D, Hodi FS, Gutzmer R, Neyns B, et al. Nivolumab versus chemotherapy in patients with advanced melanoma who progressed after anti-CTLA-4 treatment (CheckMate 037): a randomised, controlled, open-label, phase 3 trial. Lancet Oncol. 2015.

10. Postow MA, Chesney J, Pavlick AC, Robert C, Grossmann K, McDermott D, et al. Nivolumab and ipilimumab versus ipilimumab in untreated melanoma. N Engl J Med 2015;372:2006-17.

11. Larkin J, Chiarion-Sileni V, Gonzalez R, Grob JJ, Cowey CL, Lao CD, et al. Combined Nivolumab and Ipilimumab or Monotherapy in Untreated Melanoma. N Engl J Med 2015; 373:23-34.

12. Robert C, Schachter J, Long GV, Arance A, Grob JJ, Mortier L, et al. Pembrolizumab versus Ipilimumab in Advanced Melanoma. N Engl J Med 2015;372:2521-32.

13. Ribas A, Puzanov I, Dummer R, Schadendorf D, Hamid O, Robert C, et al. Pembrolizumab versus investigator-choice chemotherapy for ipilimumab-refractory melanoma (KEYNOTE-002): A randomised, controlled, phase 2 trial. Lancet Oncol 2015;16:908-18.

14. Kottschade LA, McWilliams RR, Markovic S, Block MS, Bisneto JV, Pham AQ, et al. The use of pembrolizumab for the treatment of metastatic uveal melanoma. . J Clin Oncol. 2015; 33.

15. Tsai KK, Loo K, Khurana N, Algazi AP, Hwang J, Sanchez R, et al. Clinical characteristics predictive of response to pembrolizumab in advanced melanoma. J Clin Oncol. 2015;33.

16. Zarour HM, Tawbi H, Tarhini AA, Wang H, Sander C, Rose A, et al. Study of anti-PD-1 antibody pembrolizumab and pegylated-interferon alfa-2b (Peg-IFN) for advanced melanoma. J Clin Oncol. 2015;33.

17. Antonia SJ, Brahmer JR, Gettinger SN, Chow LQM, Juergens RA, Shepherd FA, et al. Nivolumab (anti-PD-1; BMS-936558, ONO-4538) in combination with platinum-based doublet chemotherapy (PT-DC) in advanced non-small cell lung cancer (NSCLC). J Clin Oncol. 2014;32.

18. Antonia SJ, Gettinger SN, Chow LQM, Juergens RA, Borghaei H, Shen Y, et al. Nivolumab (anti-PD-1; BMS-936558, ONO-4538) and ipilimumab in first-line NSCLC: Interim phase I results. J Clin Oncol 2014;32.

19. Rizvi NA, Mazieres J, Planchard D, Stinchcombe TE, Dy GK, Antonia SJ, et al. Activity and safety of nivolumab, an anti-PD-1 immune checkpoint inhibitor, for patients with advanced, refractory squamous non-small-cell lung cancer (CheckMate 063): a phase 2, single-arm trial. Lancet Oncol 2015;16:257-65.

20. Garon EB, Rizvi NA, Hui R, Leighl N, Balmanoukian AS, Eder JP, et al. Pembrolizumab for the treatment of non-small-cell lung cancer. N Engl J Med 2015;372:2018-28.

21. Brahmer J, Reckamp KL, Baas P, Crino L, Eberhardt WE, Poddubskaya E, et al. Nivolumab versus Docetaxel in Advanced Squamous-Cell Non-Small-Cell Lung Cancer. N Engl J Med 2015;373:123-35.

22. Gettinger SN, Horn L, Gandhi L, Spigel DR, Antonia SJ, Rizvi NA, et al. Overall Survival and Long-Term Safety of Nivolumab (Anti-Programmed Death 1 Antibody, BMS-936558, ONO- 4538) in Patients With Previously Treated Advanced Non-Small-Cell Lung Cancer. J Clin Oncol 2015;33:2004-12.

23. Borghaei H, Paz-Ares L, Horn L, Spigel DR, Steins M, Ready NE, et al. Nivolumab versus Docetaxel in Advanced Nonsquamous Non-Small-Cell Lung Cancer. N Engl J Med 2015;373:1627-39

24. Spigel DR, Chaft JE, Gettinger SN, Chao BH, Dirix LY, Schmid P, et al. Clinical activity and safety from a phase II study (FIR) of MPDL3280A (antiPDL1) in PD-L1-selected patients with non-small cell lung cancer (NSCLC). J Clin Oncol 2015;33.

25. Nishio M, Hida T, Nakagawa K, Sakai H, Nogami N, Atagi S, et al. Phase II studies of nivolumab (anti-PD-1, BMS-936558, ONO-4538) in patients with advanced squamous (sq) or nonsquamous (non-sq) non-small cell lung cancer (NSCLC). J Clin Oncol 2015;33.

26. Spira AI, Park K, Mazières J, Vansteenkiste JF, Rittmeyer A, Ballinger M, et al. Efficacy, safety and predictive biomarker results from a randomized phase II study comparing MPDL3280A vs docetaxel in 2L/3L NSCLC (POPLAR). J Clin Oncol 2015;33.

27. Goldberg SB, Gettinger SN, Mahajan A, Herbst RS, Chiang AC, Tsiouris AJ, et al. Activity and safety of pembrolizumab in patients with metastatic non-small cell lung cancer with untreated brain metastases. J Clin Oncol 2015;33.

28. Amin A, Plimack ER, Infante JR, Ernstoff MS, Rini BI, McDermott DF, et al. Nivolumab (anti-PD-1; BMS-936558, ONO-4538) in combination with sunitinib or pazopanib in patients (pts) with metastatic renal cell carcinoma (mRCC). J Clin Oncol 2014;32.

29. Choueiri TK, Fishman MN, Escudier BJ, Kim JJ, Kluger HM, Stadler WM, et al. Immunomodulatory activity of nivolumab in previously treated and untreated metastatic renal cell carcinoma (mRCC): Biomarker-based results from a randomized clinical trial. J Clin Oncol 2014;32.

30. Motzer RJ, Rini BI, McDermott DF, Redman BG, Kuzel TM, Harrison MR, et al. Nivolumab for Metastatic Renal Cell Carcinoma: Results of a Randomized Phase II Trial. J Clin Oncol. 2014.

31. McDermott DF, Choueiri TK, Puzanov I, Hodi S, Drake CG, Brahmer JR, et al. Survival, durable response, and long-term safety in patients with previously treated advanced renal cell carcinoma receiving nivolumab. J Clin Oncol 2015;33:2013-20.

32. Motzer RJ, Escudier B, McDermott DF, George S, Hammers HJ, Srinivas S, et al. Nivolumab versus Everolimus in Advanced Renal-Cell Carcinoma. N Engl J Med 2015;373:1803-1333.

33. Hammers HJ, Plimack ER, Infante JR, Rini BI, McDermott DF, Ernstoff M, et al. Expanded cohort results from checkmate 016: A phase I study of nivolumab in combination with ipilimumab in metastatic renal cell carcinoma (mRCC). J Clin Oncol 2015;33.

34. McDermott DF, Sosman JA, Sznol M, Massard C, Gordon MS, Hamid O, et al. Atezolizumab, an Anti-Programmed Death-Ligand 1 Antibody, in Metastatic Renal Cell Carcinoma: Long- Term Safety, Clinical Activity, and Immune Correlates From a Phase Ia Study. J Clin Oncol 2016; 34(8):833-42.

35. Powles T, Eder JP, Fine GD, Braiteh FS, Loriot Y, Cruz C, et al. MPDL3280A (anti-PD-L1) treatment leads to clinical activity in metastatic bladder cancer. Nature 2014;515:558-62.

36. Hamanishi J, Mandai M, Ikeda T, Minami M, Kawaguchi A, Murayama T, et al. Safety and Antitumor Activity of Anti-PD-1 Antibody, Nivolumab, in Patients With Platinum-Resistant Ovarian Cancer. J Clin Oncol. 2015; 33(34):4015-22.

37. Disis ML, Patel MR, Pant S, Infante JR, Lockhart AC, Kelly K, et al. Avelumab (MSB0010718C), an antiPDL1 antibody, in patients with previously treated, recurrent or refractory ovarian cancer: A phase Ib, openlabel expansion trial. J Clin Oncol 2015;33.

38. Varga A, Piha-Paul SA, Ott PA, Mehnert JM, Berton-Rigaud D, Johnson EA, et al. Antitumor activity and safety of pembrolizumab in patients (pts) with PDL1 positive advanced ovarian cancer: Interim results from a phase Ib study. J Clin Oncol 2015;33.

39. Berger R, Rotem-Yehudar R, Slama G, Landes S, Kneller A, Leiba M, et al. Phase I safety and pharmacokinetic study of CT-011, a humanized antibody interacting with PD-1, in patients with advanced hematologic malignancies. Clin Cancer Res 2008;14:3044-51.

40. Armand P, Nagler A, Weller EA, Devine SM, Avigan DE, Chen YB, et al. Disabling immune tolerance by programmed death-1 blockade with pidilizumab after autologous hematopoietic stem-cell transplantation for diffuse large B-cell lymphoma: results of an international phase II trial. J Clin Oncol 2013;31:4199-206.

41. Westin JR, Chu F, Zhang M, Fayad LE, Kwak LW, Fowler N, et al. Safety and activity of PD1 blockade by pidilizumab in combination with rituximab in patients with relapsed follicular lymphoma: a single group, open-label, phase 2 trial. Lancet Oncol 2014;15:69-77.

42. Ansell SM, Lesokhin AM, Borrello I, Halwani A, Scott EC, Gutierrez M, et al. PD-1 blockade with nivolumab in relapsed or refractory Hodgkin's lymphoma. N Engl J Med 2015;372: 311- 9.

43. Lesokhin AM, Ansell SM, Armand P, Scott EC, Halwani A, Gutierrez M, et al. Preliminary results of a phase I study of nivolumab (BMS-936558) in patients with relapsed or refractory lymphoid malignancies. Blood. 2014;124.

44. Doi T, Piha-Paul SA, Jalal SI, Mai-Dang H, Yuan S, Koshiji M, et al. Pembrolizumab (MK3475) for patients (pts) with advanced esophageal carcinoma: Preliminary results from KEYNOTE028. J Clin Oncol 2015;33.

45. Brahmer JR, Drake CG, Wollner I, Powderly JD, Picus J, Sharfman WH, et al. Phase I study of single-agent anti-programmed death-1 (MDX-1106) in refractory solid tumors: safety, clinical activity, pharmacodynamics, and immunologic correlates. J Clin Oncol. 2010; 28: 3167-75.

46. Brahmer JR, Tykodi SS, Chow LQ, Hwu WJ, Topalian SL, Hwu P, et al. Safety and activity of anti-PD-L1 antibody in patients with advanced cancer. N Engl J Med 2012;366:2455-65.

47. Topalian SL, Hodi FS, Brahmer JR, Gettinger SN, Smith DC, McDermott DF, et al. Safety, activity, and immune correlates of anti-PD-1 antibody in cancer. N Engl J Med 2012;366:2443- 54.

48. Herbst RS, Soria JC, Kowanetz M, Fine GD, Hamid O, Gordon MS, et al. Predictive correlates of response to the anti-PD-L1 antibody MPDL3280A in cancer patients. Nature 2014;515:563- 7.

49. Le DT, Uram JN, Wang H, Bartlett BR, Kemberling H, Eyring AD, et al. PD-1 Blockade in Tumors with Mismatch-Repair Deficiency. N Engl J Med 2015;372:2509-20.

50. Patnaik A, Kang SP, Rasco D, Papadopoulos KP, Elassaiss-Schaap J, Beeram M, et al. Phase I Study of Pembrolizumab (MK-3475; Anti-PD-1 Monoclonal Antibody) in Patients with Advanced Solid Tumors. Clin Cancer Res 2015;21:4286-93.

51. Plimack ER, Bellmunt J, Gupta S, Berger R, Montgomery RB, Heath K, et al. Pembrolizumab (MK-3475) for advanced urothelial cancer: Updated results and biomarker analysis from KEYNOTE012. J Clin Oncol 2015;33.
